# Suprachoroidal Triamcinolone Acetonide for the Treatment of Refractory Macular Edema Secondary to Non-Infectious Uveitis

**DOI:** 10.3390/pharmaceutics18060675

**Published:** 2026-05-29

**Authors:** Bryant Menke, Charlene H. Choo, Marc Ohlhausen, Timothy Kaftan, Nam Nguyen, Lindsay Helget, Alan Erickson, Christopher D. Conrady, Steven Yeh

**Affiliations:** 1 Truhlsen Eye Institute, Department of Ophthalmology, University of Nebraska Medical Center, Omaha, NE 68105, USAcchoo@unmc.edu (C.H.C.);; 2 Department of Internal Medicine, Division of Rheumatology, University of Nebraska Medical Center, Omaha, NE 68198, USA; 3 Department of Pathology and Microbiology, University of Nebraska Medical Center, Omaha, NE 68198, USA; 4 Global Center for Health Security, University of Nebraska Medical Center, Omaha, NE 68198, USA

**Keywords:** suprachoroidal triamcinolone acetonide, Xipere, uveitis, macular edema

## Abstract

**Background/objective:** Suprachoroidal triamcinolone acetonide (TA) was recently FDA-approved and is emerging as a new alternative to other local therapies for macular edema (ME) associated with noninfectious uveitis (NIU). The objective of this study is to evaluate the preliminary safety and efficacy of suprachoroidal TA in patients with refractory ME secondary to NIU. **Methods:** This was a retrospective review of a small cohort of patients with refractory ME secondary to NIU treated with suprachoroidal TA from November 2022 to October 2023. **Results:** Six eyes from five patients with refractory ME secondary to NIU were included in the study. The cohort included two females (40%), and the median age was 62 years (IQR = 8). Ophthalmic diagnoses included intermediate uveitis (*n* = 2; 40%), birdshot chorioretinopathy (*n* = 1; 20%), autoimmune retinopathy (*n* = 1; 20%), and panuveitis (*n* = 1; 20%). The median logMAR visual acuity was 0.7 (Snellen 20/100) at baseline and improved to 0.3 (Snellen 20/40) during follow-up visits at 1 month and 2–3 months. The median central subfield thickness (CST) was 690 μm at baseline and improved to 367.5 μm and 309 μm at the follow-up visits at 1 month and 2–3 months, respectively. The initial improvement in logMAR visual acuity and CST was less pronounced at follow-up visits at 6–7 months and 11–12 months. **Conclusions**: This study demonstrates the safety of suprachoroidal TA and efficacy signals, including improvement in visual acuity and ME at 3 months in patients with severe, refractory ME secondary to NIU.

## 1. Introduction

Macular edema (ME) is the most common complication of uveitis and a major cause of visual impairment in patients with chronic uveitis [1,2]. Systemic and locally administered corticosteroids are the mainstay of treatment for noninfectious uveitis (NIU) complicated by ME, but a recent survey study of uveitis and retina specialists found that local therapy was preferred for unilateral or posterior segment disease [3]. Commonly used local therapies included intravitreal dexamethasone implant (Ozurdex^®^), periocular triamcinolone acetonide (TA), and intravitreal TA. In the Multicenter Uveitis Steroid Treatment (MUST) clinical trial, patients with intermediate, posterior, and panuveitis who were treated with sustained-release fluocinolone acetonide (FA) implants had comparable visual outcomes to those treated with systemic corticosteroids [4]. However, a local corticosteroid implant was associated with a higher risk of elevated intraocular pressure (IOP) and cataract surgery. This was followed by the PeriOcular versus INTravitreal corticosteroids for uveitis macular edema (POINT) trial that compared the safety and efficacy of various forms of local corticosteroid therapy [5]. The results of the POINT study showed that intravitreal TA and dexamethasone implants were superior to periocular TA in improving or resolving ME associated with uveitis but were associated with a higher risk of elevated IOP.

Suprachoroidal injection is a novel local drug delivery method that targets the potential space between the sclera and choroid. The favorable pharmacokinetics include higher levels of the therapeutic agent within posterior segment layers, including the choroid and retina, and lower levels of the agent in anterior segment structures with the potential for greater efficacy and reduced adverse events, including cataract and IOP [6]. The favorable pharmacokinetic profile of suprachoroidal drug delivery has prompted investigations for its use in various diseases affecting the posterior segment, including ME secondary to diabetes, retinal vein occlusion, uveitis, age-related macular degeneration, and choroidal melanoma [7]. Recent clinical trials have demonstrated the safety and efficacy of suprachoroidal injection of TA suspension in patients with ME secondary to NIU over a period of up to 48 weeks. In 2021, suprachoroidal injection of TA suspension CLS-TA (Xipere^TM^) was FDA-approved for the treatment of ME associated with uveitis [8].

In this study, we evaluated the preliminary safety and efficacy of suprachoroidal TA in a limited cohort of patients with refractory ME secondary to NIU. Our results support favorable vision and optical coherence tomography (OCT) outcomes during short-term follow-up with a potential need for adjunctive agents over time in patients with more refractory inflammatory eye disease.

## 2. Materials and Methods

This study was a retrospective review of patients with refractory ME secondary to NIU who were treated with suprachoroidal corticosteroid injections from 2021 to 2023 at the Truhlsen Eye Institute, University of Nebraska Medical Center (UNMC). Refractory ME was defined as persistent edema ≥ 2 months despite treatment with at least one systemic immunomodulatory agent for ≥1 month of treatment duration and at least one of the following local therapies: corticosteroid-releasing intravitreal implant, Subtenon’s corticosteroid injection, or topical corticosteroid drops. Approval for this study was obtained from the Institutional Review Board at UNMC. Patients were treated according to the best medical judgment of providers following standard-of-care practices with appropriate consultations from other services as clinically indicated. The Standardization of Uveitis Nomenclature was used to classify the anatomic location of uveitis, including anterior, intermediate, posterior, and panuveitis [9]. Systemic therapies were individualized by patient characteristics, uveitis subtype classification, established treatment paradigms, and multidisciplinary care discussions.

Data were collected on demographic information, ophthalmic history, and exam and imaging findings at baseline visit and during follow-up within 1 month, 2–3 months, 6–7 months, and 11–12 months. OCT scans were reviewed for central subfield thickness (CST), defined as the central 1 mm zone with automated segmentation software (Version 11.5) on the Zeiss Cirrus 5000^TM^ spectral-domain OCT (Carl Zeiss, Oberkochen, Germany). CST was measured with calipers in images with segmentation errors. For adverse events following suprachoroidal injections, data were collected on IOP and cataract development in addition to serious adverse events, such as retinal detachment, endophthalmitis, or serious illness requiring hospitalization.

Snellen visual acuities (VA) were converted to logarithm of the minimum angle of resolution (logMAR) values [10]. Descriptive and inferential statistical analyses were performed with median VA reported along with the interquartile ranges (IQRs) at the designated follow-up time points. The Wilcoxon signed-rank test was used to compare the median logMAR VA, CST, and IOP from baseline to each follow-up visit. Eyes with light perception vision or worse were excluded from analysis (*n* = 1), as these values cannot be reliably converted to logMAR visual acuities. Primary outcomes were median change in logMAR VA from baseline to 11–12 months follow-up and median change in CST from baseline to 11–12 months follow-up. Safety outcomes included the incidence of steroid-related adverse events such as significantly increased IOP and progression of cataract from baseline to follow-up visits.

Generative artificial intelligence was not utilized in any phase of this study or in the writing of this manuscript.

## 3. Results

This study included five patients with refractory ME secondary to NIU who were treated with suprachoroidal TA (Table 1). The cohort had a median age of 62 years (IQR = 8) and included two females (40%). All patients were white or Caucasian (100%). The ophthalmic diagnoses included intermediate uveitis (*n* = 2; 40%), birdshot chorioretinopathy with positive HLA-A29 (*n* = 1; 20%), autoimmune retinopathy (*n* = 1; 20%), and panuveitis (*n* = 1; 20%). All patients were either receiving or had previously trialed systemic anti-inflammatory therapy at the time of suprachoroidal TA administration. The most common previous systemic treatment included oral prednisone (*n* = 3; 60%) and methotrexate (*n* = 2; 40%). Two patients (40%) were on oral prednisone at the time of suprachoroidal TA administration. One of these patients was also on methotrexate (20%) and adalimumab (20%). One patient was on cyclosporine (20%) at the time of suprachoroidal TA administration.

Six eyes from five patients with refractory ME secondary to NIU were treated with suprachoroidal TA (Table 2). Four out of six eyes were pseudophakic (66.7%). At the baseline visit, the median logMAR VA was 0.7 (IQR = 0.17), and the median CST was 690 μm (IQR = 568). The most common previous local treatment was intravitreal steroid implant (*n* = 5; 83.3%), followed by topical corticosteroids (*n* = 3; 50.0%) and periocular triamcinolone acetonide injection (*n* = 3; 50%). One patient had a previous suprachoroidal TA injection within the last two months. At the time of suprachoroidal TA injection, two eyes (40%) from two patients were being treated with topical corticosteroids.

**Figure 1:**
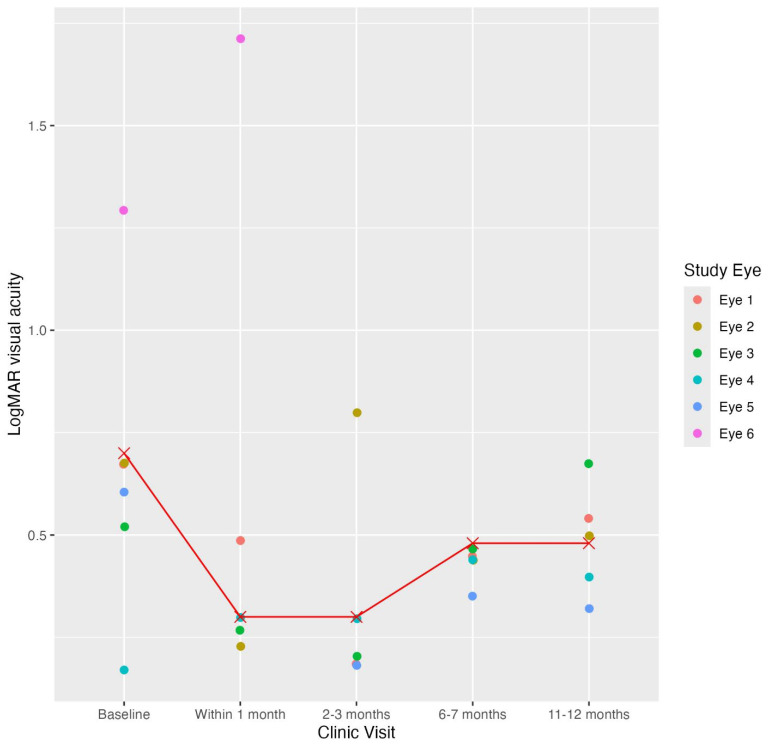
LogMAR visual acuity of eyes at baseline and follow-up visits. The scatter plot includes the logMAR visual acuity of each eye treated with suprachoroidal triamcinolone acetonide at baseline and follow-up visits. Median logMAR visual acuity at baseline and each follow-up visit is depicted by a red cross mark connected by a line graph.

**Figure 2:**
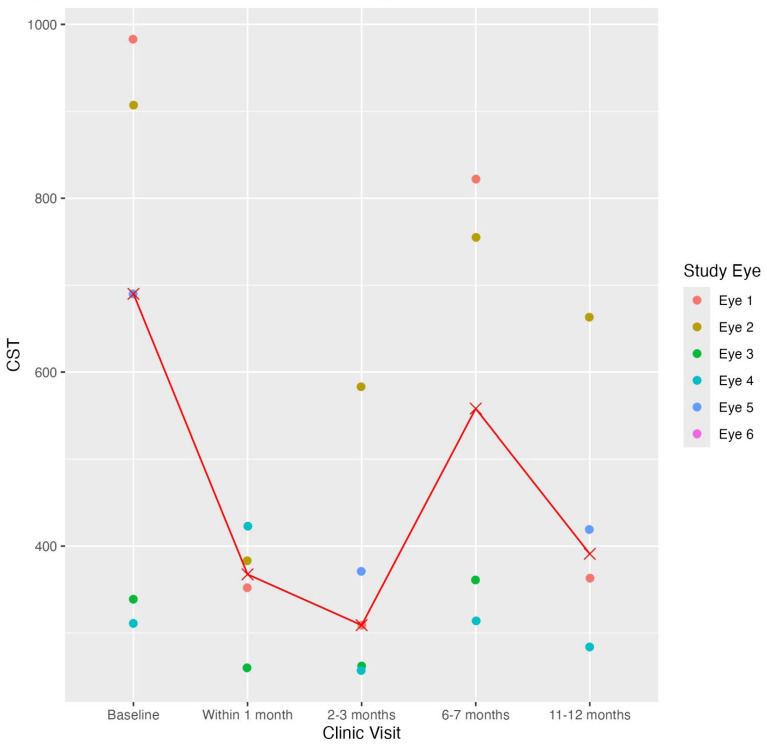
Central subfield thickness of eyes at baseline and follow-up visits. The scatter plot includes the central subfield thickness (CST) of each eye treated with suprachoroidal triamcinolone acetonide at baseline and follow-up visits. Median CST at baseline and each follow-up visit is depicted by a red cross mark connected by a line graph.

**Figure 3:**
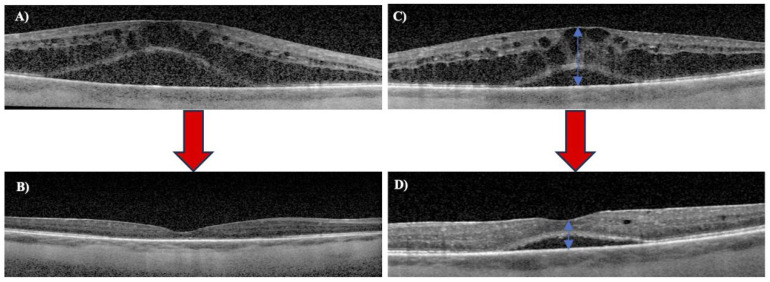
An example of the resolution of macular edema following suprachoroidal triamcinolone acetonide (TA) injection. Optical coherence tomography (OCT) image of the right eye (**A**) and left eye (**C**) showing intraretinal and subretinal fluid prior to suprachoroidal TA injection. Blue arrows represent an example measurement of central subfield thickness. (**B**) OCT of the right eye with resolution of intraretinal and subretinal fluid 7 weeks after suprachoroidal TA injection. (**D**) OCT of the left eye with significant improvement of intraretinal and subretinal fluid 5 weeks after suprachoroidal TA injection.

The median logMAR VA was 0.7 (IQR = 0.165) at the baseline visit, 0.3 (IQR = 0.24) at the follow-up within 1 month, 0.3 (IQR = 0.12) at 2–3 months, 0.48 (IQR = 0.14) at 6–7 months, and 0.48 (IQR = 0.18) at 11–12 months (Table 3; Figure 1). The median CST was 690 μm (IQR = 568) at the baseline visit, 367.5 μm (IQR = 64) at the follow-up within 1 month, 309 μm (IQR = 109) at 2–3 months, 771.8 μm (IQR = 422.5) at 6–7 months, and 391 μm (IQR = 136.8) at 11–12 months (Figure 2 and Figure 3). The median IOP was 14 mmHg (IQR = 11) at the baseline visit, 11 mmHg (IQR = 6) at the follow-up within 1 month, 14.5 mmHg (IQR = 11.8) at 2–3 months, 11 mmHg (IQR = 18) at 6–7 months, and 14.5 mmHg (IQR = 5) at 11–12 months. VA improved by two or more lines in two out of five eyes (40.0%) and remained stable in three out of five eyes (60.0%) at the follow-up within 1 month and 2–3 months. Four out of five eyes (80%) showed stable VA or improvement of two or more lines at 11–12-month follow-up, while one patient demonstrated vision loss from Snellen 20/100 at baseline to 20/200 at this follow-up time point. There was no statistically significant difference between the median logMAR VA and CST at the baseline and follow-up visits (*p* > 0.05).

The median IOP was not significantly elevated during the follow-up period compared to the baseline visit (*p* > 0.05). Two out of six phakic eyes (33.3%) did not show evidence of worsening cataracts after suprachoroidal TA injection. There were no serious adverse events, including retinal detachment, endophthalmitis, or serious illness requiring hospitalization.

## 4. Discussion

In this small cohort of patients with refractory ME secondary to NIU, the median logMAR VA and CST improved at follow-up visits within 1 month and 2–3 months after administration of suprachoroidal TA. While the improvement in VA and OCT outcomes was notable in several patients, statistical analyses of the improvement in both measures were limited by the small sample size. Efficacious endpoints of median change in VA and CST at 11–12-month follow-up were not statistically significant from baseline. In addition, a ceiling effect for improvement in VA may also be observed in patients with severe disease, given that concomitant improvement in CST was observed when a clear VA benefit was not observed. The trends in CST were similar to the findings observed in the PEACHTREE phase III clinical trial that compared the efficacy of suprachoroidal TA injection to a sham procedure in patients with ME secondary to NIU [11]. Specifically, improvement in OCT outcomes was observed at 1-, 2-, and 3-month follow-up, at which time a second suprachoroidal injection was administered in the PEACHTREE trial. The PEACHTREE trial results showed that the median time to rescue therapy was 89 days in the treatment arm compared to 36 days in the control arm, suggesting that the effects of suprachoroidal TA may last at least 12 weeks. A recent retrospective study by Panse and colleagues that investigated the effect of suprachoroidal TA in 51 patients with NIU also found that almost 50% of eyes required additional treatment for ME at the 12-week follow-up [12]. Of note, within the MAGNOLIA study that evaluated the need for rescue therapy in a subset of patients from PEACHTREE who did not require rescue therapy, 50% of patients did not require rescue therapy at the 48-week follow-up visit, approximately 9 months after the second suprachoroidal injection in the PEACHTREE study [13].

Concurrent systemic medication remained constant in 4/5 patients throughout 11–12 months of follow-up. One patient started 5 mg oral prednisone during the 11–12-month follow-up period. All patients underwent tapers of topical corticosteroids as clinically indicated throughout the follow-up period. The need for individualized treatment plans and variable timing of topical corticosteroids represents a potential confounding factor in CST response.

Our retrospective data showed reduced CST until the follow-up at 2–3 months but some recurrence of ME during follow-up visits at 6–7 months and 11–12 months. Similar to the PEACHTREE study, these findings indicate reduced therapeutic effects over time, which likely represent temporal drug clearance rather than loss of treatment effectiveness. This is supported by PEACHTREE participant treatment response seen after receiving a second suprachoroidal injection. Encouraging safety endpoints observed include lack of cataract progression in 2/2 phakic eyes (100%), and IOP was not significantly different between the baseline and follow-up visits. Further studies are needed to evaluate the effect of suprachoroidal TA in patients with refractory ME secondary to NIU and long-term adverse events associated with repeat treatment.

The POINT trial was a multicenter, randomized clinical trial that investigated 8-week post-treatment CST following periocular TA, intravitreal TA, or intravitreal dexamethasone (Ozurdex) implant in the setting of uveitic macular edema [5]. Similar to our findings, all three treatment groups experienced meaningful CST reductions at 8-week follow-up compared to baseline. In contrast to our study, the POINT trial found a modest increase in the risk of IOP elevation among all treatment groups. While our cohort size limits statistical analysis, the observed safety endpoints suggest a potentially favorable IOP profile of suprachoroidal administration; however, additional comparative studies are required.

In addition to suprachoroidal TA treatment, all patients were concurrently treated with immunomodulatory therapies of varying targets of action, including anti-metabolites (methotrexate), T-cell calcineurin inhibitors (cyclosporine), and tumor necrosis factor-alpha (TNF-α) inhibitors (adalimumab). These patients required laboratory monitoring and were jointly managed with colleagues in Rheumatology, highlighting the importance of multidisciplinary care paradigms and targeting both systemic and local inflammation in the care of complex uveitis patients.

Anti-metabolites and anti-TNF- α therapies are recommended in moderate to severe intermediate NIU, posterior NIU, and pan-NIU. In practice, selection of the systemic agents involves a nuanced consideration of specific patient characteristics, treatment history, and multidisciplinary care discussions. One patient with a history of branch retinal vein occlusion (BRVO) showed no improvement following a year of treatment with adalimumab. Given the inadequate response to anti-TNF-α therapy and intravitreal steroid and anti-VEGF injections, the patient was treated with suprachoroidal TA. Subsequent follow-up revealed reduced CST and improved VA. Two other patients received systemic corticosteroid therapy without corticosteroid-sparing agents. In one of these patients, corticosteroid-sparing immunomodulatory therapy was deferred due to considerable comorbidities and polypharmacy. Another patient with NIU had a retinal vasoproliferative tumor and underwent a series of intraocular steroid implants, Subtenon’s steroid injections, and anti-VEGF injections prior to treatment with suprachoroidal injection, which was associated with signs of controlled local inflammation.

Limitations included the absence of a control group and the retrospective nature of the study in a limited cohort of patients with refractory ME at a tertiary care, university-based institution. Patients had follow-up visits as clinically indicated, so data were not collected according to a study protocol. Additionally, due to patient complexity, conventional treatment recommendations were, at times, not advised. Therefore, in these instances, the persistence of ME may be reflective of inadequate anti-inflammatory coverage rather than refractory disease. Uveitis is a heterogeneous collection of over 30 diseases involving intraocular inflammation [14]. Inclusion of multiple uveitis subtypes may introduce confounding factors such as subtype-specific treatment responses, concurrent treatments variations, and expected disease course. Although the improved clinical findings observed in patients across varying uveitis subtypes may indicate support for suprachoroidal TA in the management of refractory ME in a multitude of uveitis syndromes, further research is required to validate these findings.

Despite these limitations, observed descriptive trends in VA and anatomic OCT outcomes lasting up to 12 weeks may support the therapeutic benefit of suprachoroidal TA for the management of refractory or potentially undertreated ME associated with NIU. While clinical metrics appeared to show improvement following suprachoroidal TA, this study was not powered to sufficiently confirm treatment efficacy or safety in patients with uveitic ME refractory to other therapies. Whether scheduled injections (i.e., two injections scheduled at day 0 and week 12 per the PEACHTREE protocol) may lead to a greater efficacy and durability signals than a single injection protocol is an important consideration given the PEACHTREE protocol that mandated suprachoroidal injections at baseline and at 3-month follow-up [11]. Further studies regarding the optimal dosing intervals for patients with severe or refractory ME associated with NIU are needed, as well as optimal treatment paradigms to manage systemic and local medications in the care of patients with complex inflammatory eye disease.

## Figures and Tables

**Table 1:** Demographic and clinical characteristics of patients with refractory ME secondary to NIU treated with suprachoroidal triamcinolone acetonide.

Characteristics	Total (*n* = 5)
Median age, years (IQR)	62 (8)
Female, *n* (%)	2 (40)
White or Caucasian race, *n* (%)	5 (100)
Ophthalmic diagnosis, *n* (%)	
Intermediate uveitis	2 (40)
Panuveitis	1 (20)
Birdshot chorioretinopathy, HLA-A29+	1 (20)
Autoimmune retinopathy	1 (20)
All previous systemic treatment, *n* (%)	
Oral prednisone	3 (60)
Immunomodulatory therapy	2 (40)
Methotrexate	2 (40)
Adalimumab	2 (40)
Azathioprine	1 (20)
Cyclosporine	1 (20)
Mycophenolate mofetil	1 (20)
Antiviral	1 (20)
* Concurrent systemic treatment, *n* (%)	
Oral prednisone	2 (40)
Immunomodulatory therapy	2 (50)
Adalimumab	2 (40)
Methotrexate	1 (20)
Cyclosporine	1 (20)

ME = macular edema, NIU = noninfectious uveitis, *n* = number of patients, IQR = interquartile range, HLA = human leukocyte antigen. * Systemic immunosuppressive therapy was co-managed with the Division of Rheumatology at UNMC.

**Table 2:** Clinical characteristics of eyes with refractory ME associated with NIU treated with suprachoroidal triamcinolone acetonide.

Characteristics	Total (*n* = 6)
Pseudophakia, *n* (%)	4 (66.7)
Baseline logMAR visual acuity, median (IQR)	0.7 (0.17)
Baseline CST in μm, median (IQR)	690 (568)
Baseline intraocular pressure in mmHg, median (IQR)	14 (11)
All previous local treatment, *n* (%)	
Topical corticosteroid	3 (50.0)
Subtenon Kenalog	3 (50.0)
Intravitreal steroid implant	5 (83.3)
Intravitreal anti-VEGF	2 (33.3)
Suprachoroidal TA	1 (16.7)
Concurrent local treatment, *n* (%)	
Topical corticosteroid	2 (66.7)

ME = macular edema, NIU = noninfectious uveitis, *n* = number of eyes, logMAR = logarithm of the minimum angle of resolution, IQR = interquartile range, CST = central subfield thickness, anti-VEGF = anti-vascular endothelial growth factor, TA = triamcinolone acetonide.

**Table 3:** LogMAR visual acuity, CST, and IOP of eyes at baseline and follow-up visits.

Clinic Visit	LogMAR VA, Median (IQR)	CST in μm, Median (IQR)	IOP in mmHg, Median (IQR)
Baseline visit	*n* = 6 0.7 (0.165)	*n* = 5 690 (568)	*n* = 6 14 (11)
Follow-up within 1 month	*n* = 5 0.3 (0.24)	*n* = 4 367.5 (64)	*n* = 5 11 (6)
Follow-up at 2–3 months	*n* = 5 0.3 (0.12)	*n* = 5 309 (109)	*n* = 5 14.5 (11.8)
Follow-up at 6–7 months	*n* = 5 0.48 (0.14)	*n* = 4 771.8 (422.5)	*n* = 5 11 (18)
Follow-up at 11–12 months	*n* = 5 0.48 (0.18)	*n* = 4 391 (136.8)	*n* = 5 14.5 (5)

LogMAR = logarithm of the minimum angle of resolution, CST = central subfield thickness, IOP = intraocular pressure, VA = visual acuity, IQR = interquartile range, *n* = number of eyes.

## Data Availability

The original contributions presented in this study are included in the article. Further inquiries can be directed to the corresponding author.

## Abbreviations

The following abbreviations are used in this manuscript:

ME	Macular edema
NIU	Noninfectious uveitis
TA	Triamcinolone acetonide
MUST	Multicenter Uveitis Steroid Treatment
FA	Fluocinolone acetonide
IOP	Intraocular pressure
POINT	PeriOcular versus INTravitreal corticosteroids for uveitis macular edema
FDA	Food and Drug Administration
OCT	Optical coherence tomography
UNMC	University of Nebraska Medical Center
CST	Central subfield thickness
VA	Visual acuity
logMAR	Logarithm of the minimum angle of resolution
IQR	Interquartile ranges
TNF-α	Tumor necrosis factor-alpha
